# The origin of projections from the posterior cingulate and retrosplenial cortices to the anterior, medial dorsal and laterodorsal thalamic nuclei of macaque monkeys

**DOI:** 10.1111/ejn.12389

**Published:** 2013-10-18

**Authors:** John P Aggleton, Richard C Saunders, Nicholas F Wright, Seralynne D Vann

**Affiliations:** 1 School of Psychology, Cardiff University70, Park Place, Cardiff, Wales, CF10 3AT, UK; 2 Laboratory of Neuropsychology, National Institute of Mental HealthBethesda, MD, 20892, USA

**Keywords:** cingulate cortex, memory, primate, retrosplenial cortex, thalamus

## Abstract

Interactions between the posterior cingulate cortex (areas 23 and 31) and the retrosplenial cortex (areas 29 and 30) with the anterior, laterodorsal and dorsal medial thalamic nuclei are thought to support various aspects of cognition, including memory and spatial processing. To detail these interactions better, the present study used retrograde tracers to reveal the origins of the corticothalamic projections in two closely related monkey species (*Macaca mulatta, Macaca fascicularis*). The medial dorsal thalamic nucleus received only light cortical inputs, which predominantly arose from area 23. Efferents to the anterior medial thalamic nucleus also arose principally from area 23, but these projections proved more numerous than those to the medial dorsal nucleus and also involved additional inputs from areas 29 and 30. The anterior ventral and laterodorsal thalamic nuclei had similar sources of inputs from the posterior cingulate and retrosplenial cortices. For both nuclei, the densest projections arose from areas 29 and 30, with numbers of thalamic inputs often decreasing when going dorsal from area 23a to 23c and to area 31. In all cases, the corticothalamic projections almost always arose from the deepest cortical layer. The different profiles of inputs to the anterior medial and anterior ventral thalamic nuclei reinforce other anatomical and electrophysiological findings suggesting that these adjacent thalamic nuclei serve different, but complementary, functions supporting memory. While the lack of retrosplenial connections singled out the medial dorsal nucleus, the very similar connection patterns shown by the anterior ventral and laterodorsal nuclei point to common roles in cognition.

## Introduction

Interest in the connections of the posterior cingulate and retrosplenial cortices with the anterior thalamic nuclei stems from electrophysiological and disconnection studies that indicate how these pathways might support learning and memory (Gabriel, 1993; Sutherland & Hoesing, 1993). Neuroimaging and neuropathological findings also reveal that both the retrosplenial cortex and anterior thalamic nuclei display abnormalities from almost the earliest stages of Alzheimer's disease (Braak & Braak, 1991a,b; Minoshima *et al*., 1997; Pengas *et al*., 2010), implying that their interconnections are particularly vulnerable. Furthermore, the laterodorsal nucleus seemingly shares functions with the anterior thalamic nuclei (Hopkins, 2005; Edelstyn *et al*., 2006; Taube, 2007), raising questions about the extent to which they share similar cortical connections. In contrast, the medial dorsal nucleus has been implicated in aspects of cognition different to those linked with the anterior thalamic or laterodorsal nuclei (e.g., Van der Werf *et al*., 2000, 2003), yet this nucleus is also interconnected with posterior cingulate cortices. All of these issues reinforce the need to detail cingulate–thalamic connections and understand their functional implications.

Projections from the posterior cingulate cortex (areas 23 and 31) and retrosplenial cortex (areas 29 and 30) terminate in the anterior thalamic nuclei, the medial dorsal thalamic nucleus, the laterodorsal nucleus and the medial pulvinar (Vogt *et al*., 1979, 1987; Baleydier & Mauguiere, 1980; Yeterian & Pandya, 1988; Morris *et al*., 1999; Shibata & Yukie, 2003, 2009; Hsu & Price, 2007). All of these studies have, however, relied on anterograde tracers. As a consequence they provide relatively crude information about the precise sources of these projections. For example, in no study is there a case with a tracer injection confined to area 29, prompting a review to note that ‘the definitive targets of corticothalamic projections from area 29 remain unknown’ (Shibata & Yukie, 2009; *P*. 97). Consequently, the sources of the projections from some posterior cingulate and retrosplenial sites have been inferred by comparing the results from overlapping, but different, cortical injections (e.g., Yeterian & Pandya, 1988).

The present study used retrograde tracers to identify the sources of posterior cingulate and retrosplenial projections to the anterior, medial dorsal and laterodorsal thalamic nuclei. Overall objectives included determining whether some thalamic nuclei have particular affinities with the retrosplenial cortex, given the direct hippocampal projections that target this cortical area in preference to the posterior cingulate cortex (Aggleton *et al*., 2012).The principal information came from eight rhesus monkeys (*Macaca mulatta*). Of these, four had injections of the same fluorescent tracer in opposite hemispheres to maximise information. This method does, however, mean that any crossed corticothalamic projections should be considered when interpreting the findings. Current evidence indicates that crossed cingulate projections are light, with the anterior medial nucleus likely to receive the majority of such inputs (Baleydier & Mauguiere, 1980; Yeterian & Pandya, 1988; Morris *et al*., 1999; Shibata & Yukie, 2003). To examine this particular issue, additional information came from three cynomolgus monkeys (*Macaca fascicularis*), each with a single injection of horseradish peroxidase in the anterior medial thalamic nucleus.

## Materials and methods

All of the injections were made in the same laboratory at the National Institute of Mental Health (Bethesda, Maryland, USA). In order to minimise the use of monkeys, the study predominantly relied on archived sections from cases prepared between ∽10 years (fluorescent tracers) and 30 years (horseradish peroxidase) previous. These cases originally came from two separate studies. One study concerned the projections from the amygdala and hippocampal formation to the medial thalamus (Aggleton & Mishkin, 1984; Aggleton *et al*., 1986), the other examined inputs from the parahippocampal region and mammillary bodies to the thalamus (Saunders *et al*., 2005; Vann *et al*., 2007). A number of recent cases (KSHU, CSR, CT8C) provided additional information and helped to validate data from these older cases.

### General procedure

Data were collected from two different series of tracer injections. One series examined the retrograde transport of two fluorescent dyes [Fast Blue (FB) and Diamidino Yellow (DY)], typically with both injected into the same animal to reduce the total number of cases. The other series (three cases) examined the retrograde transport of horseradish peroxidase (HRP). Despite minor variations in some of the experimental procedures, as well as possible differences in the sensitivity of the various tracers, both series of tracer injections are described as they complement one another and so help to validate the findings. All experimental procedures were carried out with strict adherence to the NIH Guide for Care and Use of Laboratory Animals, such that the ‘Principles of Laboratory Animal Care’ (NIH Publication No. 86-23, revised 1985) were followed. The study and its procedures were approved by the National Institute of Mental Health (NIMH, Bethesda, Maryland, USA).

### Fluorescent tracer injections

Eight adult rhesus monkeys received injections of the fluorescent tracers DY (Keizer *et al*., 1983) and FB (Kuypers *et al*., 1980). In six cases the two tracers were targeted at different thalamic sites in the same animal (Table1). The animals were first tranquilized with ketamine hydrochloride (10–15 mg/kg, intramuscular injection) and surgical anaesthesia maintained with gas (isoflurane 1–4%, to effect). The monkeys were placed in a specialized head holder and, following aseptic procedures, dorsal bone and dural flaps were opened to expose the midline. The wall of the left hemisphere was then gently retracted and a 5- to 10-mm portion of the corpus callosum and the underlying fornix were split longitudinally to expose the thalamic midline. Under visual guidance, an injection of either FB (Sigma, St Louis, MO, USA) or DY (Sigma) was made through a 5-μL Hamilton syringe fitted with a 28-gauge needle. Both tracers were injected as 3% suspensions in distilled water. Following tracer injection, the dura and skin were sutured. All animals were given prophylactic antibiotics (claforan) starting the day before surgery, as well as during the surgery (i.v.) and for 7–10 days after surgery. Post-operative analgesia was provided by either ketprofen or buprenorphine, as determined by veterinary guidance. Immediately following surgery, as each animal began to wake, it was placed in a heated recovery cage in which humidity and oxygen levels were controlled. In all cases recovery was without incident. All procedures described were included on an NIMH-approved animal study protocol.

**Table 1:** List of all cases

Case	Sp	Injection site	Ipsilateral		
			Surgery	Tracer	Hemisphere
ACy1	Cy	AM Mid Re (MD)	None	HRP	
ACy2	Cy	AM Mid	None	HRP	
ACy26	Cy	AM Re	None	HRP	
KSHU	Rh	MD	None	FB	Right
CSR	Rh	MD	None	DY	Left
CT8C	Rh	MD	None	FB	Right
CT8C	Rh	AM AV (AD)	None	DY	Left
BRh2	Rh	AM	None	FB	Right
		MD Mid	None	DY	
BRh3	Rh	AM (AV)	FnX	DY	Right
		MD	FnX	FB	Right
		AM	Amyg/TSX	FB	Left
		MD	Amyg/TSX	DY	Left
BRh4	Rh	AV (AM)	FnX	FB	Right
		MD	FnX	DY	Right
		AM (Cdc)	Amyg/TSX	DY	Left
		MD	Amyg/TSX	FB	Left
BRh5	Rh	AV (AM)	None	FB	Left
		LD	None	FB	Right
		LD	None	DY	Left
BRh6	Rh	LD	FnX	DY	Right
		MD (CM PCN)	FnX	FB	Right
		Mid	Amyg/TSX on left	DY	

The Table shows the species (Sp) of macaque (Rh, rhesus; Cy, cynomolgus), principal injection site (AD, anterior dorsal nucleus; AM, anterior medial nucleus; AV anterior dorsal nucleus; Cdc, nucleus centralis densocellularis; CM, nucleus centrum medianum; LD, laterodorsal nucleus; MD medial dorsal nucleus; Mid, midline thalamic nuclei; PCN, nucleus paracentralis; Re, nucleus reuniens), any other surgery in same hemisphere as injection (Amyg/TSX, amygdala and temporal stem cut; FnX, fornix transection), type of tracer (DY, Diamidino Yellow; FB, Fast Blue; HRP, horseradish peroxidase) and hemisphere of injection site (where not specified, injection primarily along midline). Injection sites in parentheses reflect limited involvement by injection.

The target sites for all of the injections, as confirmed by histology, are given in Table1. It can be seen that some cases received two injections of the same fluorescent tracer, but in different sites and in different hemispheres. In addition, in cases BRh3, BRh4 and BRh6, a fibre pathway (either the fornix or the ventroamygdalofugal pathway and adjacent temporal stem immediately lateral to the amygdala) was cut immediately before the tracer injections, i.e., in the course of the same surgery (Table1). These additional surgeries, which made it possible to test the pathways used by projections from the medial temporal lobe to the thalamus, have been described previously (Saunders *et al*., 2005; Vann *et al*., 2007). It is assumed that these additional surgeries are unlikely to have interfered with the patterns of cingulate label seen following medial thalamic injections. This assumption is based on the lack of fornical connections to and from the retrosplenial or posterior cingulate cortices in the monkey brain (Poletti & Cresswell, 1977; Mufson & Pandya, 1984; Saunders & Aggleton, 2007; Aggleton *et al*., 2012). Likewise, the ventral amygdalofugal pathway–temporal stem surgeries in the temporal lobe were far removed from any routes directly linking cingulate cortices with the thalamus. Additional checks were made by looking for any specific changes in label in those cases with tract surgery (Table2).

**Table 2:** Density of retrograde label

Case	Sp	Main injection site	Tracer	Whether other injections	Cingulate label by area					
					29	30	23a	23b	23c	31
CT8C	Rh	**MDmc**	FB	None	0	^*^	+	+	^*^	^*^
KSHU	Rh	**MDmc** rostral	FB	None	^*^	+	+	+	^*^	+
BRh2	Rh	**MDmc**	DY	None	+	+	+	+	^*^	^*^
CSR	Rh	**MDmc MDpc** rostral Cdc	DY	None	^*^	+	++	++	+	+
BRh6	Rh	**MDmc MDpc** PCN CM	FB	None	+	+	++	++	++	++
BRh3	Rh	**MDmc**	FB	R (AM)	^*^	+	+	+	+	^*^
BRh3	Rh	**MDmc**	DY	L (AM)	^*^	^*^	+	+	^*^	^*^
BRh6	Rh	**LD**	DY	R (Mid)	+++	+++	++	++	+	+
BRh5	Rh	**LD**	FB	R (AV)	++++	+++	++	+	+	+
BRh2	Rh	**AM**	FB	None	+	++	+++	+++	++	+
BRh3	Rh	**AM**	DY	R (MD)	+	++	++	++	++	+
BRh3	Rh	**AM**	FB	L (MD)	+	++	++	++	+	+
BRh4	Rh	**AM** Cdc	DY	L (MD)	+	+	++	++	+	+
ACy2	Cy	**AM mid**	HRP	None	0	^*^	++	++	0	0
ACy26	Cy	**AM mid Re**	HRP	None	+	++	+++	+++	+	^*^
ACy1	Cy	**AM Re Cdc** MD	HRP	None	+	+++	+++	+++	+	+
BRh4	Rh	**AV**AM	FB	R (PCN)	+++	+++	++	++	+	+
BRh5	Rh	**AV**AM	FB	L (LD)	++++	++++	++	++	+	+
CT8C	Rh	**AV AM AD Cdc**	DY	None	++++	++++	+++	++	++	+

The Table shows the case descriptor, species (Sp) of macaque (Rh, rhesus; Cy, cynomolgus), centre of injection site (in bold), type of tracer (DY, diamidino yellow; FB, fast blue; HRP, horseradish peroxidase), Left or Right hemisphere (L, R), site of any other injections with the same tracer in the opposite hemisphere (in parentheses), and location of cingulate label in hemisphere of injection site. The term ‘None’ refers to those cases with just one injection of a given tracer. The density of retrograde label is marked on a scale from zero to ++++. The symbol + indicates just an average of 2–5 cells per section in each area, while sporadic label (i.e., even less) is marked with ^*^. The absence of label is indicated with a 0. AD, anterior dorsal thalamic nucleus; AM, anterior medial thalamic nucleus; AV, anterior ventral thalamic nucleus; Cdc, nucleus centralis densocellularis; CM, centrum medianum; LD, laterodorsal thalamic nucleus; mc, magnocellular; MD, medial dorsal thalamic nucleus; Mid, midline thalamic nuclei; pc, parvocellular; PCN, paracentral nucleus; Re, nucleus reuniens.

After a postoperative period of between 5 and 10 days, the animals were deeply anaesthetized with sodium pentobarbital. They were then perfused intracardially with saline followed by ∽2 L of 4–6% paraformaldehyde in 0.1 m cacodylate buffer (pH 7.4). The brains were then removed and placed in a series of cryoprotectant solutions consisting of first 10% and then 20% glycerol in 0.1 m cacodylate buffer with 2% dimethylsulfoxide and 4–6% paraformaldehyde (pH 7.4, 4 °C). Four to 6 days after the perfusion, the brains were rapidly frozen by immersion in −75 °C isopentane, and then cut at 40 μm in the coronal plane on a freezing microtome (Rosene *et al*., 1986). Three series of sections (one-in-ten) were mounted immediately onto gelatine-subbed slides, dried, coverslipped and stored in the dark at 4 °C.

### HRP injections

A single injection was made into the medial thalamus in three adult cynomolgus monkeys (ACy1, ACy2, ACy26) weighing 3.5–6.8 kg at the time of surgery. The surgical procedures used to visualise the dorsal thalamus closely matched those for the fluorescent tracer injections. All injections were made under visual guidance after the corpus callosum and fornix had been divided at the midline. In all three cases the injection tract was close to the thalamic midline at the level of the anterior thalamic nuclei.

The largest HRP injection, which was in case ACy1, involved a single stereotaxic injection of 0.22 μL (40% HRP; Sigma, type VI) delivered via a 1-μL Hamilton syringe. Case ACy2 received a single injection of 0.13 μL of 40% HRP, while in case ACy26 a stereotaxic injection of 0.09 μL of a 4% solution of HRP (Sigma, type VI), conjugated with wheat germ agglutin, was targeted at the anterior thalamic region. Following injection of the tracer, the dura and skin were sutured. Prophylactic doses of antibiotics were administered to prevent infection (Bicillin, 6000 000 U), while dexamethasone phosphate (0.3 mg/kg) was given immediately after surgery to reduce any cerebral oedema. The analgesic morphine (1–2 mg/kg s.c. every 4 h) was given according to NIMH veterinary guidance, and recovery was without incident. Two days after surgery the monkeys were deeply anaesthetized with Nembutal and perfused intracardially with 0.9% saline followed by a solution of 1% paraformaldehyde and 1.25% glutaraldehyde in 0.1 m phosphate buffer (pH 7.2). The brains were stored in 30% sucrose buffer at 4 °C for 3–4 days and then cut in 50-μm coronal sections. A one-in-five series was then treated with tetramethyl benzidine according to the protocol of Hardy & Heimer (1977). Alternate sections were dehydrated, counterstained with thionin and coverslipped, while the remaining sections were just dehydrated and coverslipped.

Although these cases were injected with HRP several decades ago, it was possible to compare the present label with drawings of the locations of labelled cells made shortly after each injection. Despite some overall reduction in signal, the distribution of HRP-positive cells appeared unaffected.

### Nomenclature

The anterior thalamic nuclei in macaque monkeys comprise three major nuclei: anterior medial, anterior ventral and anterior dorsal. Immediately caudal are the laterodorsal nucleus and the medial dorsal nucleus. The medial dorsal nucleus has been subdivided into four parts: pars magnocellularis, pars parvocellularis, pars multiformis and pars densocellularis Olszewski (1952). At the level of the medial dorsal nucleus lie a series of small midline thalamic nuclei, some of which are thought to be connected with the cingulate cortices (Baleydier & Mauguiere, 1980; Yeterian & Pandya, 1988; Shibata & Yukie, 2003). The present study adopts the nomenclature and borders of these midline thalamic nuclei as determined by Olszewski (1952).

**Figure 1:**
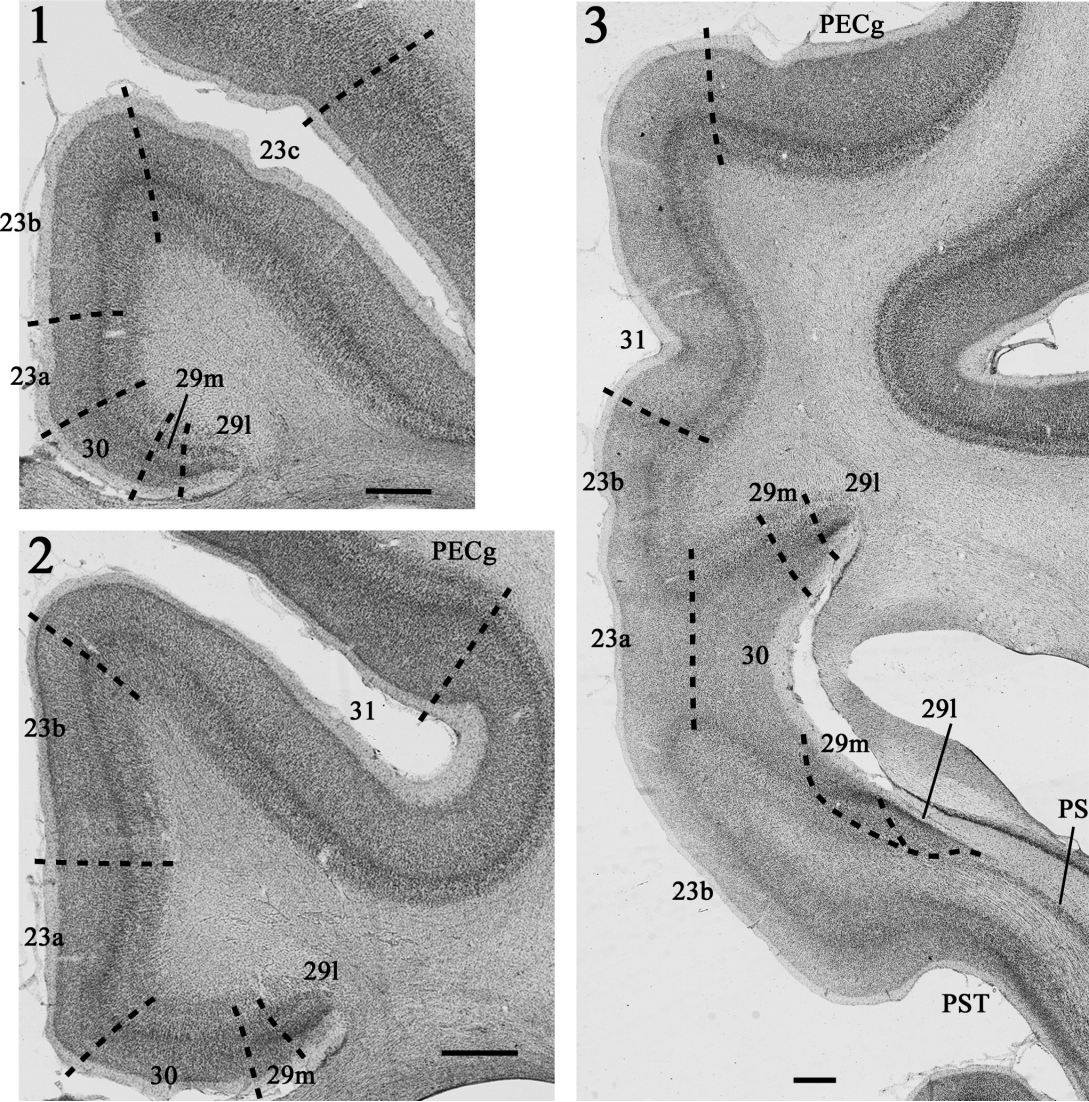
Photomicrographs (1–3) of Nissl stained coronal sections from a rhesus monkey (*Macaca mulatta*) showing the location and arrangement of the various areas within the posterior cingulate and retrosplenial cortices (areas 23, 29, 30 and 31). At more rostral levels (1 and 2) the posterior cingulate cortex is entirely dorsal to the corpus callosum. Caudal to the splenium (3) both the posterior cingulate and retrosplenial cortices extend ventrally below the splenium. PECg, parietal area PE, cingulate portion; PS, prosubiculum; PST, prostriate cortex. Scale bars, 1.0 mm.

Area 23 (Fig.1) has a rostral border with area 24, distinguished by the loss of layer IV in area 24 (Vogt *et al*., 1987). There is a transitional dysgranular area (23 d) at this border (Vogt *et al*., 2005). Area 23 has been subdivided into areas 23a, b and c (Vogt *et al*., 1987; Morris *et al*., 1999; Shibata & Yukie, 2003; Paxinos *et al*., 2009). In some studies a further distinction has been made between posterior dorsal and posterior ventral areas 23b, partly on grounds of connectivity (Shibata & Yukie, 2003; Vogt *et al*., 2005). These subareas of 23b are caudal to the splenium. Where ventral 23b reaches the most rostral part of the calcarine sulcus it borders the rostral prostriate cortex (Paxinos *et al*., 2009), a region sometimes designated 30v (Kobayashi & Amaral, 2000). The subdivisions 23a, 23b and 23c are partly determined by the width of layers II–IV, which become broader going from 23a to 23c (Vogt *et al*., 1987; Morris *et al*., 1999). Area 31, which forms the caudal and dorsal-most part of the posterior cingulate cortex, occupies much of the cingulate sulcus when area 23c disappears (Fig.1) and has even broader layers II–IV (Vogt *et al*., 1987). As the cingulate sulcus becomes more infolded, area 31 is bordered dorsally by area PECg (Paxinos *et al*., 2009).

Together, areas 29 and 30 comprise the retrosplenial cortex. The border of area 30 with area 23a is marked by the loss of a readily visible layer IV in area 23a, which is replaced by the remains of the granulous layer III(IV) in area 30 (‘dysgranular’), as well as the greater cell density found in layer Va of area 23 (Vogt, 1993; Vogt *et al*., 2005). Area 30 is distinguished from area 29 by the gradual loss of the ‘granular’ layer III(IV), along with the beginnings of a layer II and the greater development of pyramidal layers III and V in area 30. Area 29 is subdivided into area 29l and area 29m (Kobayashi & Amaral, 2000; Vogt *et al*., 2005). Area 29l is further from the midline, i.e., lateral, while area 29 m is the more medial (Fig.1). Their border is marked by the appearance of an additional layer III in area 29 m (Vogt *et al*., 1987, 2005; Morris *et al*., 1999). These same two subdivisions have also been designated areas 29a-c (equivalent to 29l) and 29d (equivalent to 29m) (Vogt *et al*., 1987; Morris *et al*., 1999; Paxinos *et al*., 2009).

## Results

The location of any retrograde label in the target region is described going from rostral to caudal, i.e., starting in rostral area 23 and finishing posterior to the splenium. Table2 provides an overview of the label found in every case as only some are described in detail. Although three monkeys also received unilateral surgical lesions of the fornix, along with transections of temporal white matter in the other hemisphere (Table1), these additional procedures had no discernible impact on retrograde tracer transport to the cingulate and retrosplenial cortices (see Table2).

### Inputs to the medial dorsal thalamic nucleus

Five cases with single tracer injections into the medial dorsal nucleus (Table1) provided the core of the information. The distribution of cingulate label was very consistent, with area 23 containing the majority of thalamic inputs, although there were differences in label density (Table2). Two additional cases with multiple thalamic injections had label profiles very similar to those seen in the other five cases (Table2) and so are not described.

**Figure 2:**
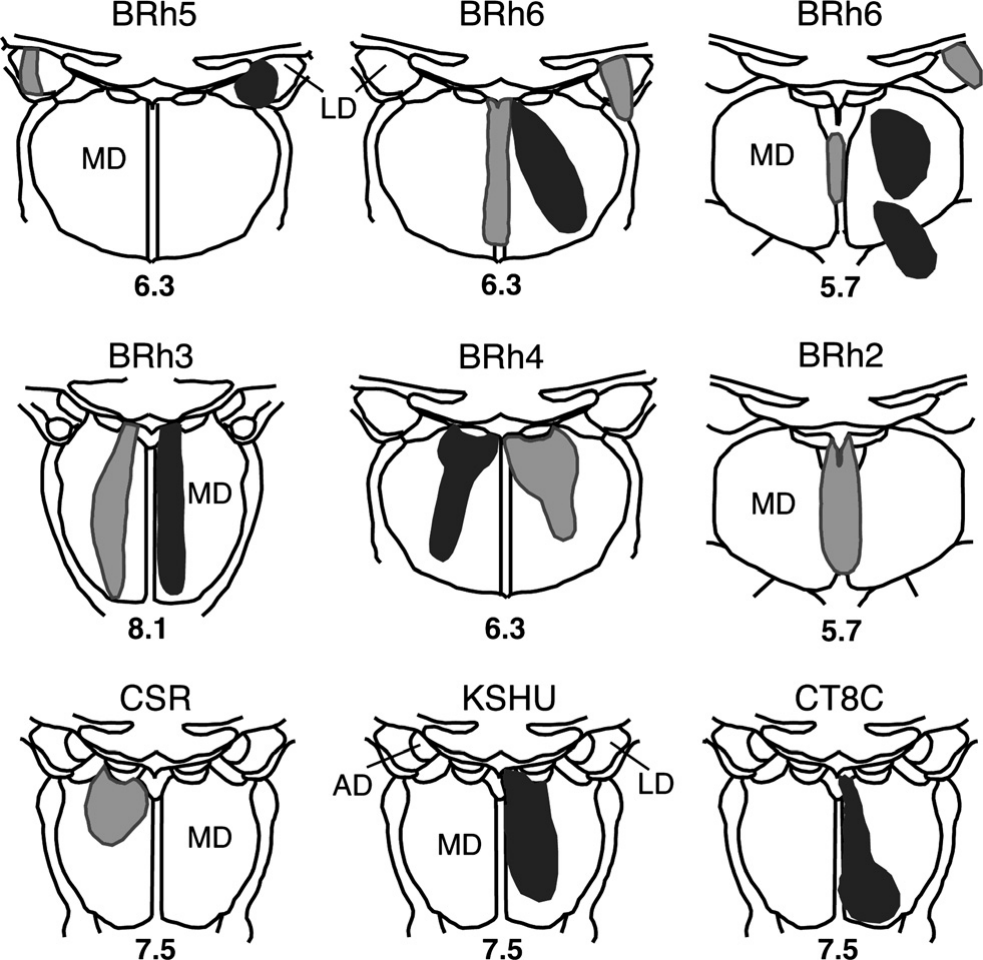
Location and extent of retrograde tracer injections involving the medial dorsal and laterodorsal thalamic nuclei. The injection sites are depicted on standard coronal sections of the medial thalamus. Each animal received an injection of FB (dark grey shading), DY (light grey shading) or both. Case numbers or names are placed above each section. The lower numbers correspond to levels depicted by Olszewski (1952), such that higher numbers are more rostral. AD, anterior dorsal thalamic nucleus; LD, laterodorsal thalamic nucleus; MD, medial dorsal thalamic nucleus.

**Figure 3:**
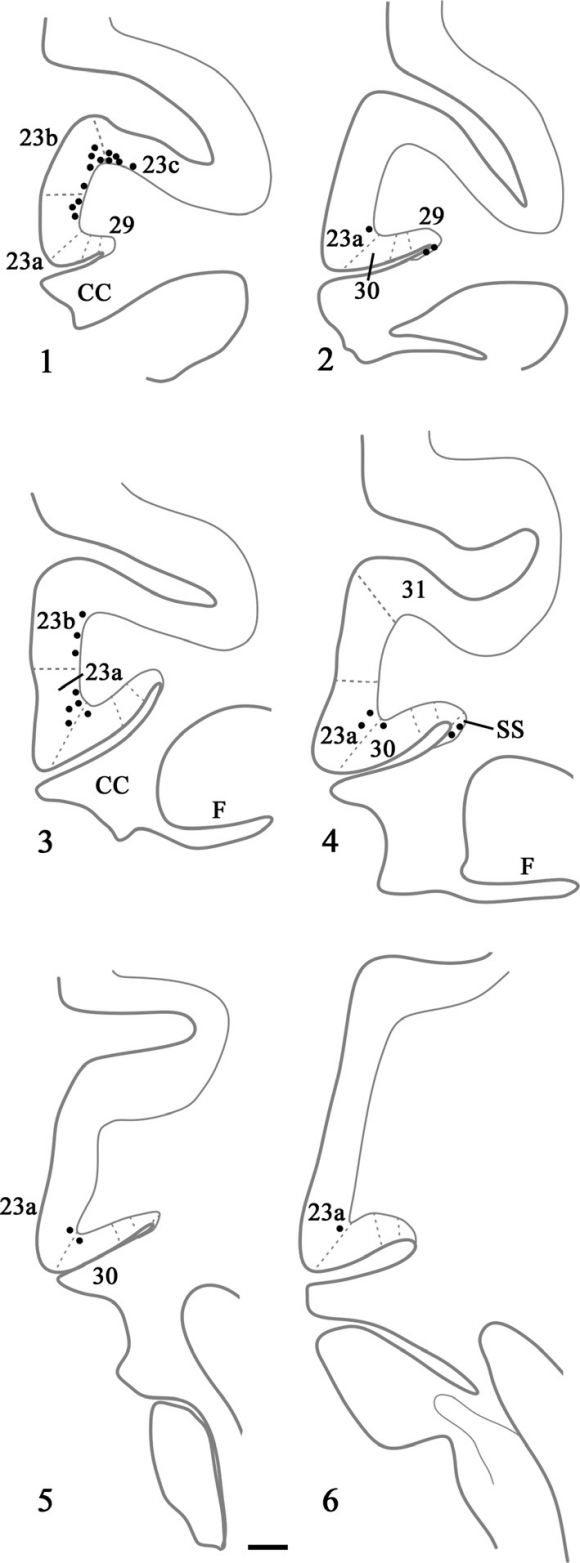
Drawings of six coronal sections in a case (CT8C) with a single injection of FB located in the magnocellular part of the medial dorsal nucleus. In this case each dot represents a single labelled cell within the cortex; 1 is the most rostral. CC, corpus callosum; F, fornix; SS, supracallosal subiculum. Scale bar, 1.0 mm.

The first three cases to be described are distinguished by the paucity of label in the posterior cingulate and retrosplenial cortices. Case CT8C had a single injection of FB (Fig.2) in the magnocellular part of the rostral medial dorsal nucleus, very close to the midline (which it appeared to reach). While most sections contained some labelled cells in the posterior cingulate cortex, the numbers were very low (almost always less than ten per section, and often less than five). These cells, which were confined to layer VI, were mainly found in area 23a and 23b (Fig.3). Elsewhere, occasional labelled cells were found in areas 23c, 30 and 31. In the contralateral hemisphere, most sections contained no FB-labelled cells, though some contained a single labelled cell (in area 23).

In case KSHU, a single injection (FB) was placed in the rostral medial dorsal nucleus (magnocellular part) with some involvement of the adjacent midline nuclei (Fig.2). A light scattering of cells was again seen across the areas of interest, with most in area 23 (as seen in case CT8C). Only one or two labelled cells were located in layer VI in each of areas 23a–c and area 30, while even less were present in area 29. Close to the splenium, a few labelled cells were present in area 31. Caudal to the splenium the few labelled cells in area 23 extended ventrally to reach the prostriate cortex (area 30v). A very small number of labelled cells were found in contralateral area 23.

In case BRh2, a single injection of DY was placed in the magnocellular part of the medial dorsal nucleus. The injection reached the immediately adjacent midline nuclei but scarcely crossed to the contralateral hemisphere (Fig.2). Label was found near the rostral border with area 24 (area 23d) and, caudal to that, every section contained DY-labelled cells in the deepest layer of areas 23a, 23b, 29 and 30, but these cells were limited in number (e.g., 10–20 cells per section in total). The label was mostly concentrated in areas 23a and 30. More caudally, labelled cells were still scattered lightly across areas 29, 30, 23a and 23b, with area 23c still containing the least within area 23, but now just a few more labelled cells were apparent in area 23b. Approaching the splenium, the posterior cingulate label remained light and the few labelled cells were typically in area 23a and adjacent area 30. Very occasional labelled cells were also found in deep areas 29m and 29l. Caudal to the splenium, the numbers of labelled cells decreased further with just a few present in areas 29l, 29m, 30 and 23b. The very rare label in the contralateral (left) hemisphere was confined to areas 23a and 23b.

**Figure 4:**
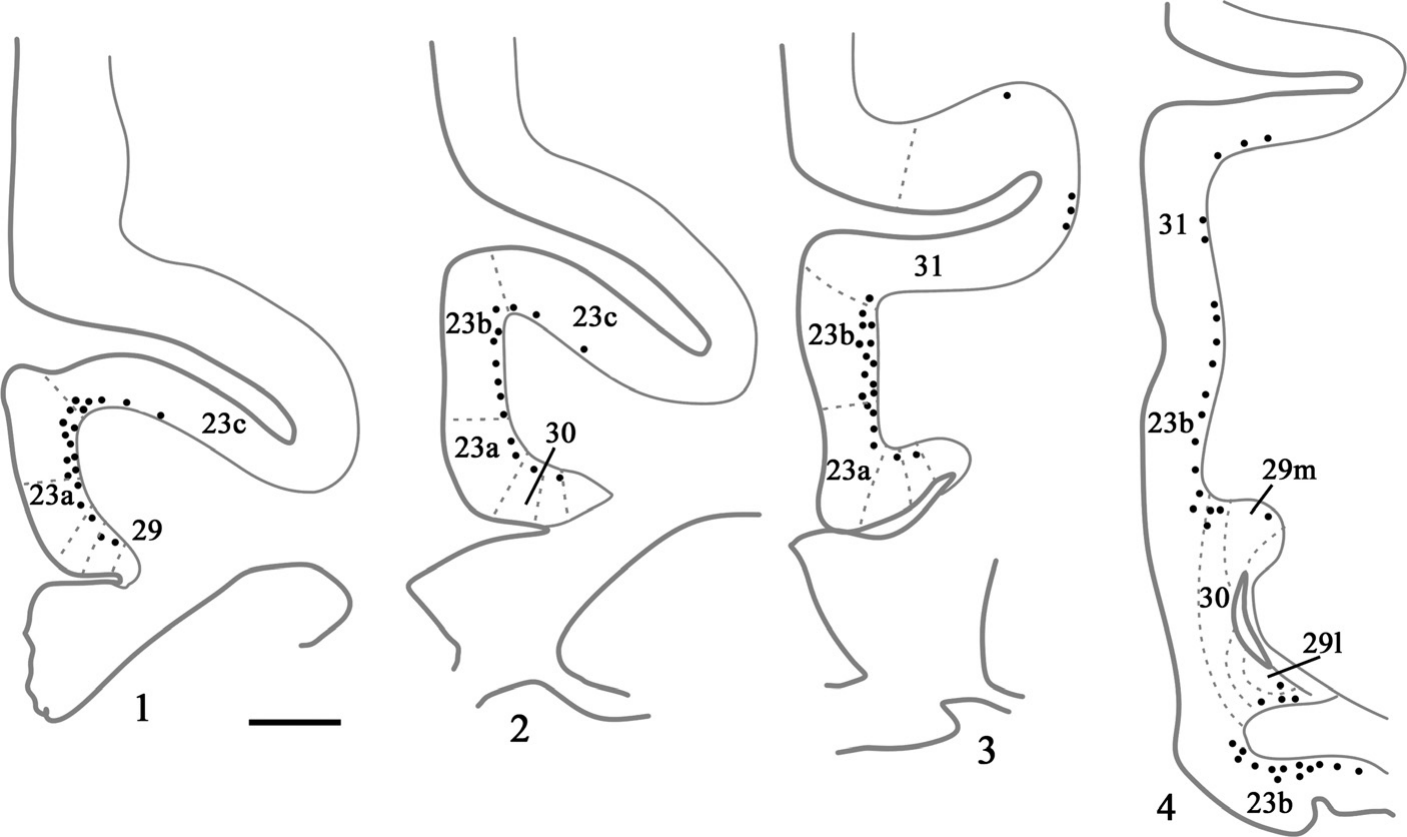
Drawings of four coronal sections depicting a case (CSR) with a single injection of DY placed in the medial dorsal nucleus. In this case each dot represents two labelled cells within the cortex. Section 4.1 is the most rostral. Scale bar, 1.5 mm.

The last two cases had appreciably more cortical label than found in the preceding three cases, although the distribution patterns were very similar. In case CSR, an injection of DY was made into the rostral medial dorsal nucleus (mainly magnocellular but also involving parvocellular; see Fig.2), though it also appeared to extend rostrally into nucleus centralis densocellularis. Labelled cells were present along the length of the region of interest, starting with area 29m. There were ∽20–60 cells in every section across the region (Fig.4). Most of the label was in layer VI of areas 23a and 23b (Fig.4). As the label extended into the cingulate sulcus to include area 23c it became sparse and often did not extend into the depths of the sulcus. Just a few labelled cells were also seen in area 29 (least in 29l), while area 30 contained a few more labelled cells than area 29. A similar light distribution of label was seen in area 31 (Fig.4). At caudal levels, labelled cells in the presubiculum formed a continuous bridge with labelled cells in the ventral retrosplenial cortex. Some label was also present in the deepest layer of the prostriate cortex. At this very caudal level, labelled cells were found in the posterior cingulate cortex on the medial wall of the hemisphere up to, and including, the ventral bank of the cingulate sulcus.

**Figure 5:**
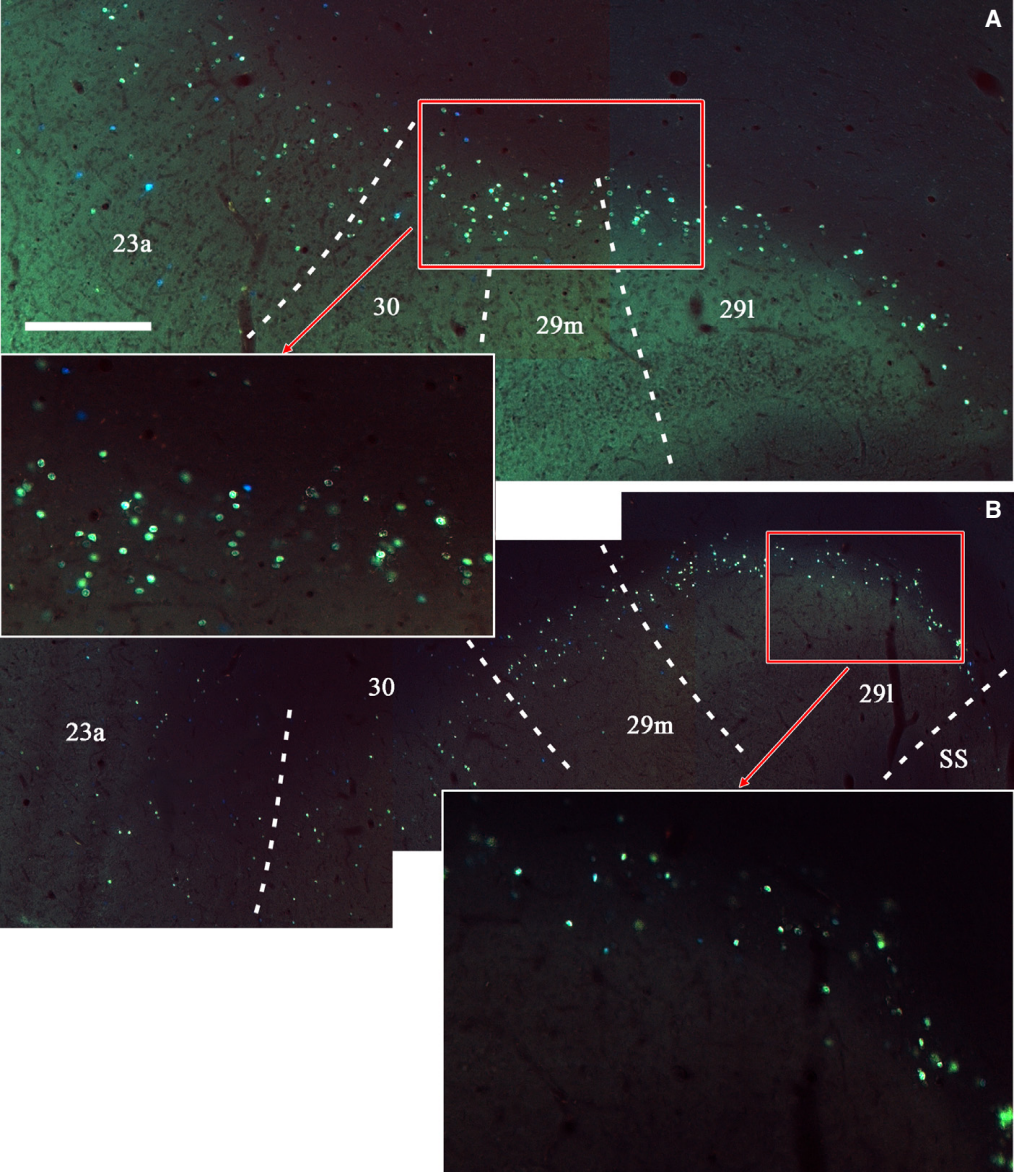
Photomicrographs of the two retrosplenial areas outlined in Fig.6 (A and B). The data, which are from Case BRh6, show the location and density of fluorescently labelled cells (DY) ipsilateral to an injection in the right laterodorsal thalamic nucleus. The two insets show specific subregions at a higher magnification. While the overwhelming majority of cells are DY (greenish yellow in appearance), there is a scattering of FB (blue) cells, especially in area 23a. These cells arise from the injections into the medial dorsal nucleus in the same hemisphere. Scale bar, 500 μm.

Finally, case BRh6 received two injections of FB involving the medial dorsal thalamic nucleus in the right hemisphere (Table1). Not only was the resulting medial dorsal nucleus injection more extensive than other cases (Fig.2), it extended into adjacent intralaminar nuclei (nucleus paracentralis and nucleus centrum medianum). This case showed an overall pattern of label similar to that described above (i.e., most cells in areas 23a and 23b, with roughly similar numbers in areas 23c, 30 and 31, and the least in area 29) except that now there were more labelled cells than seen in any of the previous cases (Table2). This increased label probably reflects both the double tracer injection and the additional involvement of adjacent intralaminar nuclei, which also receive inputs from areas 24, 23 and 30 (Baleydier & Mauguiere, 1980; Yeterian & Pandya, 1988; Shibata & Yukie, 2003). [Photomicrographs from this same case (right hemisphere) are included in Fig.5, which primarily shows label following an injection of DY into the laterodorsal nucleus. FB-labelled cells (medial dorsal injection) are visible in areas 23a, 29 and 30 in Fig.5 (especially in area 23a), though these cells are far less frequent than the DY (lime green)-labelled cells.]

In summary, only light or very light ipsilateral inputs arose from the posterior cingulate and retrosplenial cortices to reach the magnocellular part of the medial dorsal thalamic nucleus (see Table2 for additional cases). Area 23 (especially areas 23a and 23b) contained the most label, while area 29 typically contained the least. Although crossed inputs from the contralateral hemisphere may exist, they are extremely few in number. In two cases (CSR, BRh6), where the injection additionally involved the parvocellular division of the medial dorsal nucleus, labelled cells were more numerous but the relative distribution of label was very similar to that in the other cases.

### Inputs to the laterodorsal thalamic nucleus

**Figure 6:**
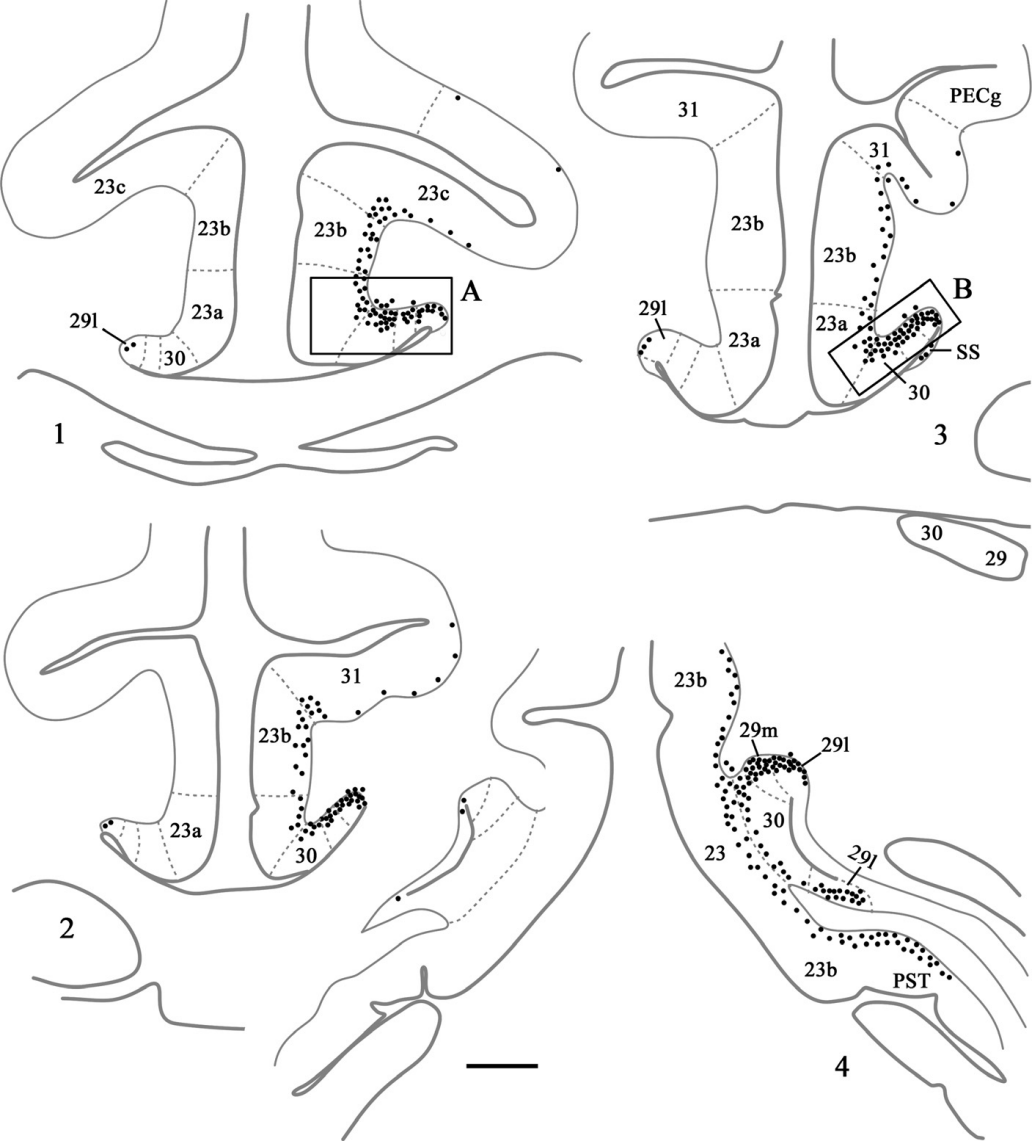
Distribution of retrograde label (Case BRh6) following an injection of DY into the laterodorsal nucleus in the right hemisphere, along with a second injection largely confined to the midline thalamic nuclei (see Fig.2). The dots reflect the relative distributions of labelled cells with each dot representing approximately three labelled cells (see Fig.5). Note the lack of labelled cells contralateral to the lateral dorsal thalamic injection. Photomicrographs of the two areas in boxes (A and B) are shown in Fig.5. PECg, parietal area; PE, cingulate portion; PST, prostriate cortex. Scale bar, 1.0 mm.

Case BRh6 received an injection of DY in the right lateral dorsal nucleus. The second DY injection in this same case was extremely restricted and essentially confined to midline thalamic nuclei (Fig.2). Given the midline location of this second injection and the scarcity of label in the left posterior cingulate and retrosplenial cortices (the very few labelled cells were confined to area 29l; see Fig.6) this second injection would add little to the overall pattern of label resulting from the laterodorsal injection in the right hemisphere (see also cases KSHU and BRh2 where midline involvement led to few, if any, labelled cells).

Ipsilateral to the laterodorsal injection, case BRh6 contained numerous labelled cells across areas 29, 30 and 23a, b and c (Fig.6). This label was most concentrated in area 29, followed by area 30 (Figs5 and 6). The quantity of label in area 23 was very similar in areas 23a and 23b, with a reduction in label only occurring in area 23c (Fig.6). That label in area 23c was mainly found near its border with 23b. In the rest of area 23c, label was very diffusely scattered in both the upper and lower banks of the cingulate sulcus, and this light label continued caudally into area 31. The posterior cingulate label was found in layer VI, with only occasional labelled cells appearing to be present in layer V of areas 23b and 23c. At more caudal levels (i.e., behind the medial dorsal nucleus) the DY label became increasingly concentrated in areas 29 and 30 (Figs5 and 6), although there were still large numbers of labelled cells spread fairly evenly across layer VI of areas 23a and 23b and, to a lesser extent, area 31. (Counts of labelled cells per section at this level gave up ∽170 cells in area 29, of which two-thirds were in 29l, ∽70 cells in area 30 and ∽30 cells each in areas 23a and 23b). Caudal to the splenium, label continued in both the dorsal and ventral retrosplenial cortex (Fig.6). Only occasional cells were seen in the contralateral cingulate or retrosplenial cortices, i.e., crossed projections were very sparse (Fig.6).

**Figure 7:**
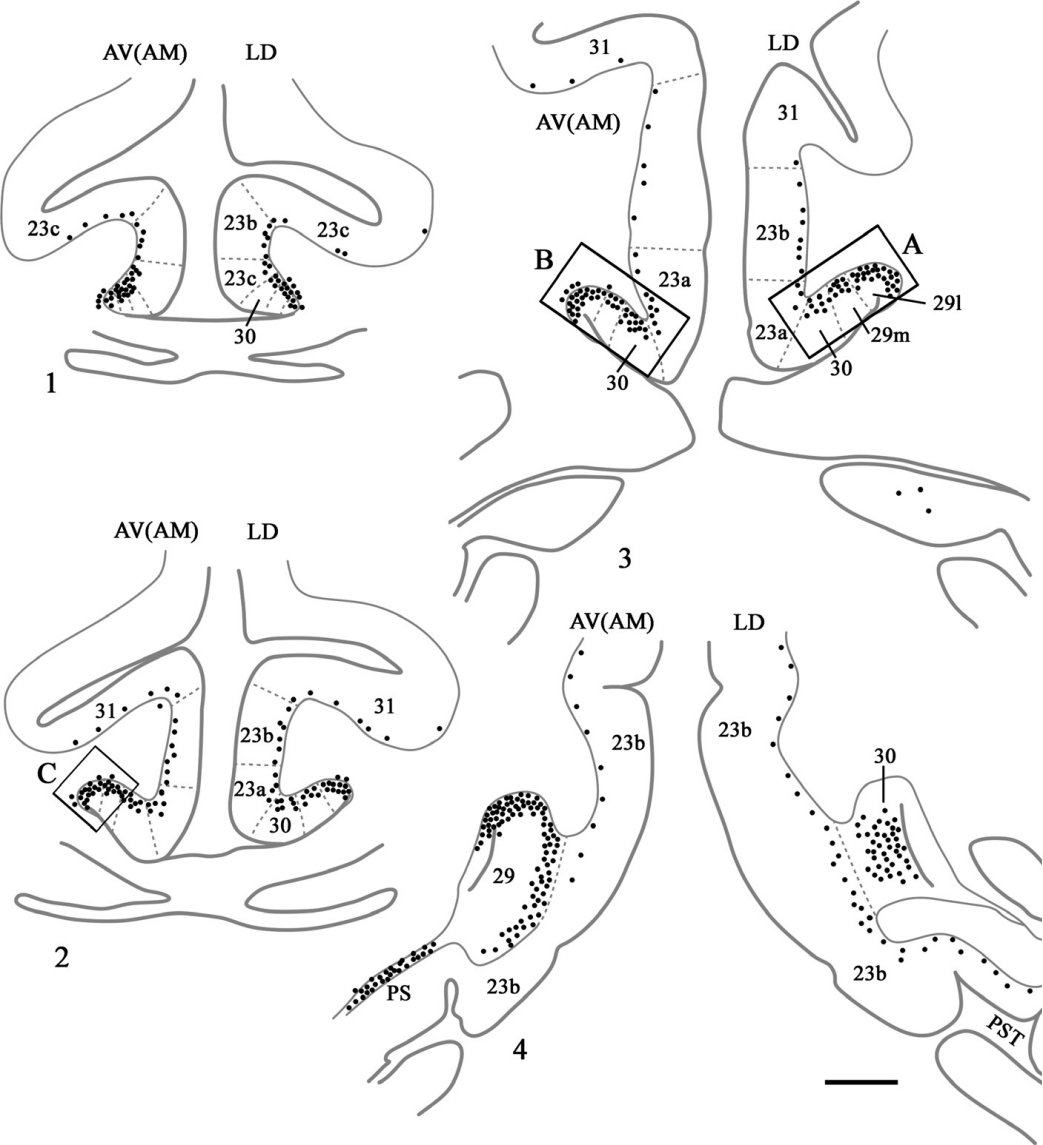
Distribution of retrograde label (Case BRh5) following an injection of FB into the caudal part of the anterior ventral nucleus in the left hemisphere, along with a second injection of FB into the laterodorsal thalamic nucleus in the right hemisphere. The dots reflect the relative distributions of labelled cells as there were too many to depict individually (see Fig.8). Photomicrographs of the three areas in boxes (A, B and C) are shown in Fig.8. PS, prosubiculum; PST, prostriate cortex. Scale bar, 2.0 mm.

**Figure 8:**
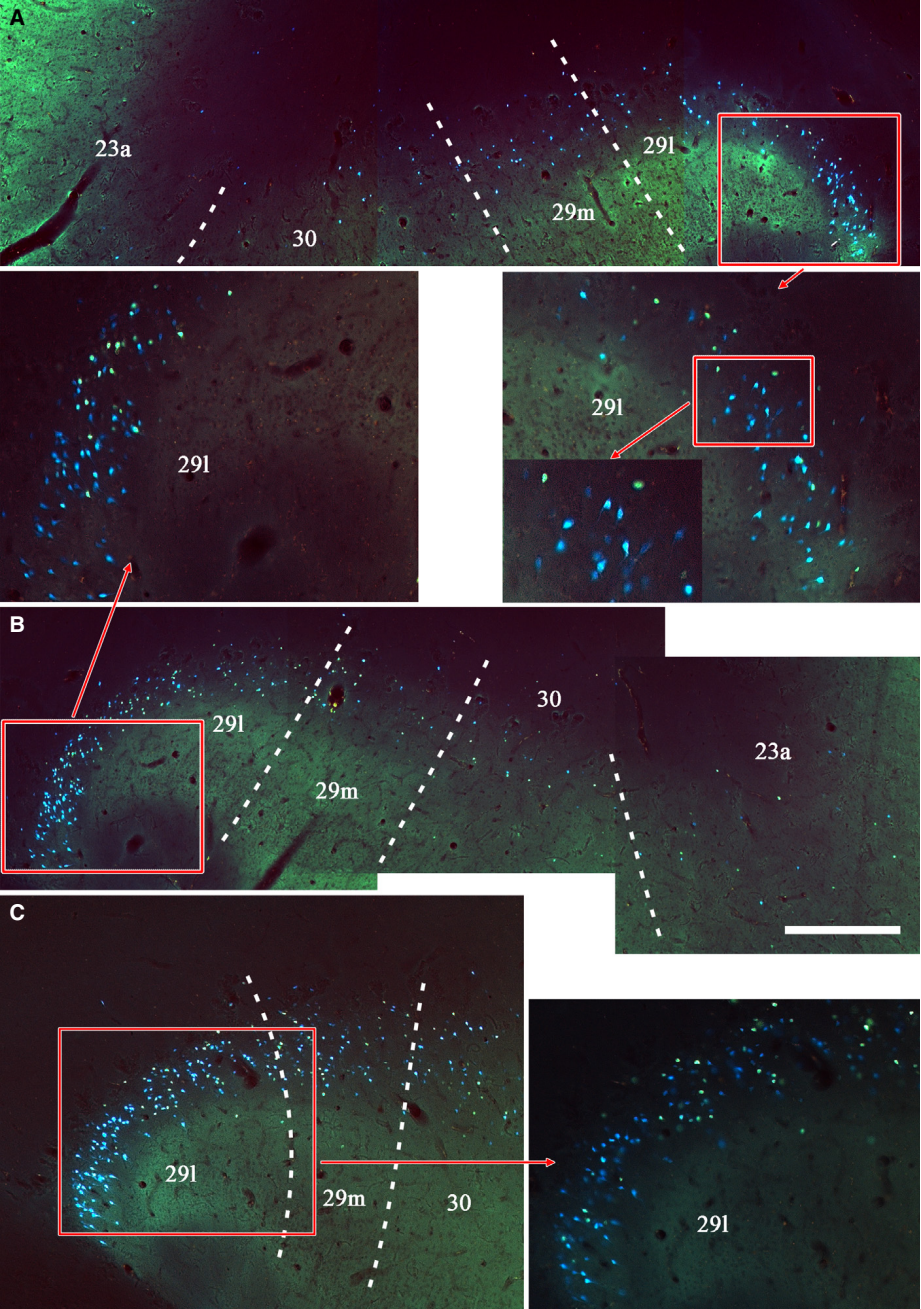
Photomicrographs from Case BRh5 showing the three areas of retrosplenial cortex identified by boxes A, B and C in Fig.7. Box A is ipsilateral to an FB injection in the laterodorsal thalamic nucleus (right hemisphere). Boxes B and C show the location and density of fluorescently labelled cells (FB) ipsilateral to an injection in the anterior ventral nucleus (left hemisphere). The insets show specific subregions at higher magnifications. While the large majority of labelled cells are blue, it can be seen that there are also numerous DY-labelled cells (lime green colour) that show a similar distribution. This additional label cells is a result of the DY injection into the lateral dorsal nucleus in the left hemisphere (not described as there is also uptake of this tracer by the corpus callosum). Scale bar, 500 μm.

In a second case (BRh5), FB was injected into the right laterodorsal nucleus (Table1). At rostral levels labelled cells were present in layer VI of areas 29, 30 and 23 (Fig.7, right) forming a clear ventral to dorsal gradient (highest concentrations in area 29, lowest in area 23c, which contained only occasional label in the lower bank and fundus; Fig.7, Box A). At more caudal levels, i.e., above the middle of the medial dorsal nucleus, the ipsilateral label was increasingly concentrated in areas 29 and 30, with few labelled cells across areas 23a and 23b (Fig.7, right). That part of 23a adjoining area 30 often contained most of the area 23 cells. Above the corpus callosum, areas 29 and 30 of BRh5 often together contained ∽100 labelled cells per section (most in area 29), while rostral area 23 would contain fewer than 10 cells in total and caudal area 23 fewer than five cells in total per section. Close to the splenium, the ipsilateral FB label became most concentrated in area 29l, although areas 29m and 30 still contained many labelled cells (Fig.7, right and Fig.8, Box A). Only at the very caudal level of areas 29 and 30, i.e., next to the fusing of the cingulate and hippocampal fissures, did the label in deep areas 29 and 30 extend ventrally to the caudomedial lobule. Here, labelled cells were also scattered across the deepest layer of area 23b. It should be noted that case BRh5 also received an injection of FB into the contralateral anterior ventral thalamic nucleus. As Baleydier & Mauguiere (1980), along with cases still to be described, found no crossed projections to the anterior ventral nucleus, the large majority of the dense ipsilateral signal presumably originated from the laterodorsal nucleus.

### Inputs to the anterior medial thalamic nucleus

**Figure 9:**
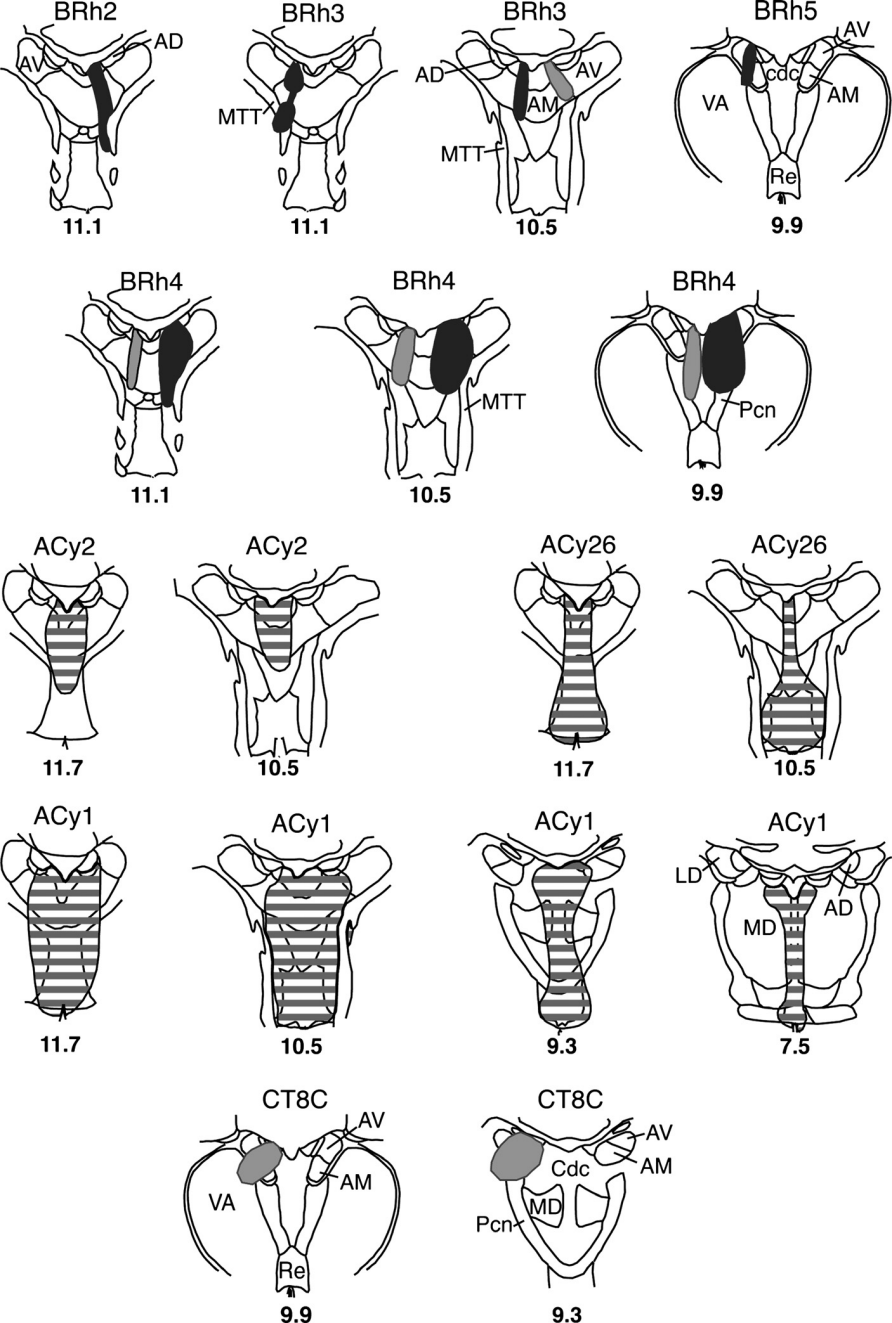
Location and extent of retrograde tracer injections involving the anterior medial and anterior ventral thalamic nuclei. The injection sites are depicted on standard coronal sections depicting the medial thalamus. Five animals received an injection of either FB (dark grey shading), DY (light grey shading) or both. Three cases received a single injection of HRP (grey horizontal lines). Case numbers or names are placed above each section. The numbers below correspond to levels depicted by Olszewski (1952), such that the higher numbers are more rostral. AD, anterior dorsal thalamic nucleus; AM, anterior medial nucleus; AV, anterior ventral nucleus; Cdc, nucleus centralis densocellularis; MD, medial dorsal thalamic nucleus; MTT, mammillothalamic tract; Pcn, nucleus paracentralis; Re, nucleus reuniens; VA, ventral anterior nucleus.

**Figure 10:**
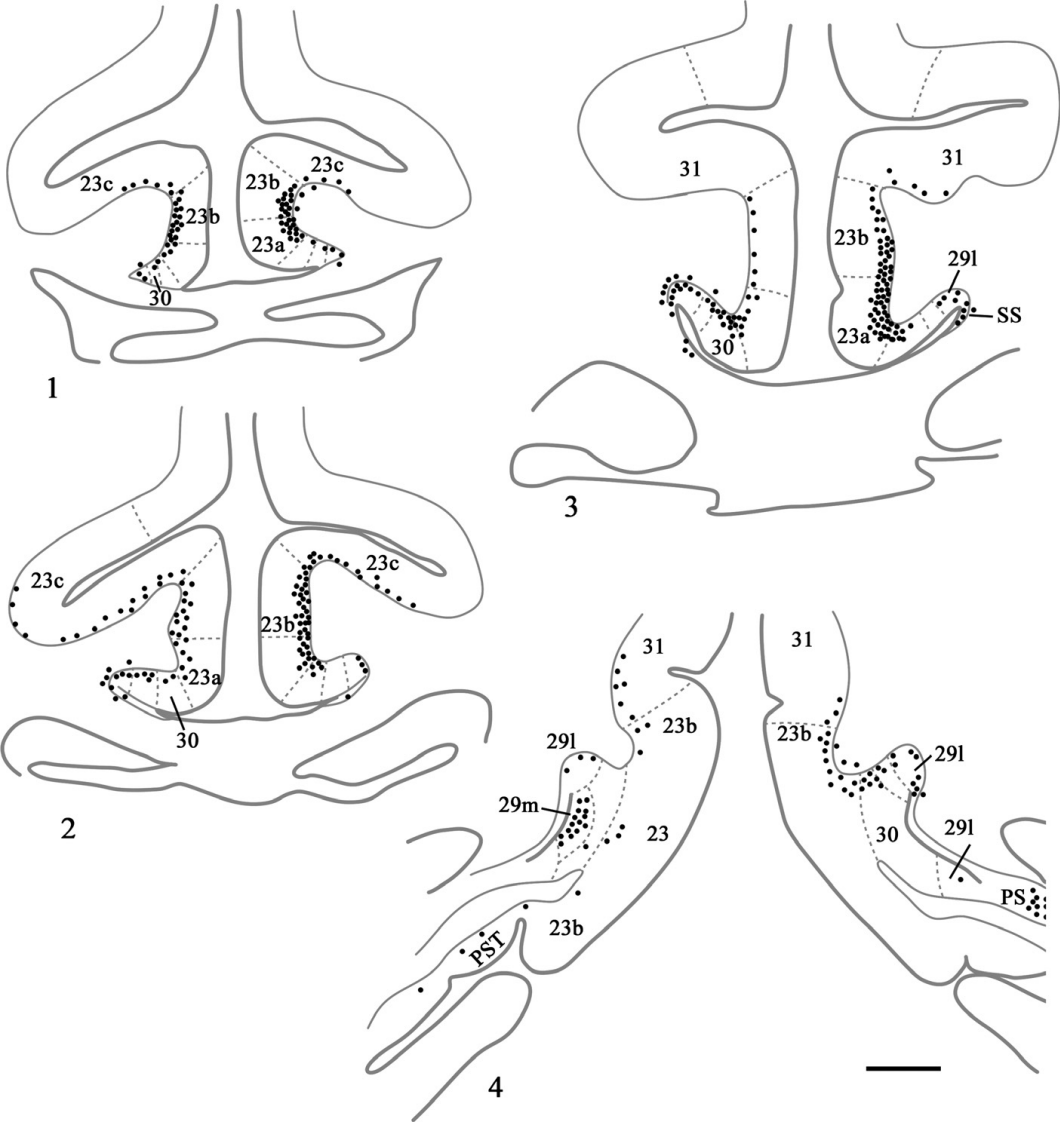
Distribution of retrograde label (Case BRh2) following an injection of FB into the midline and anterior medial thalamic nucleus in the right hemisphere (with minor involvement of the left hemisphere). The dots reflect the relative distributions of labelled cells as there were too many to depict individually (see text). Note the appreciable numbers of labelled cells contralateral to the anterior medial thalamic injection. PS, presubiculum; PST, prostriate cortex; SS, supracallosal subiculum. Scale bar, 2.0 mm.

FB was injected into the right anterior medial nucleus in case BRh2 (Fig.9), although it appeared to involve immediately adjacent parts of the paraventricular nucleus and parataenial nucleus. Appreciable fluorescent label was seen bilaterally in the posterior cingulate and retrosplenial cortices (Fig.10). While most label was in the injection hemisphere, the distribution of label was very similar in the two hemispheres (Fig.10). Ipsilateral to the injection, large numbers of labelled cells were present in the posterior cingulate cortex, starting from its rostral border in area 23d, and then in the remainder of area 23 (areas 23a, 23b and medial 23c; Fig.10). This label was essentially confined to layer VI, with occasional cells in layer V. Retrograde label was also found in areas 29 (29l and 29m) and 30, but this label was consistently less dense than that in area 23. At this rostral level ∽100 labelled cells were found per section, with most in areas 23a and 23b. This same pattern continued going caudally through the posterior cingulate and retrosplenial areas above the corpus callosum, with area 23b often containing the highest concentration of label (Fig.10). There was, however, evidence of a gradual decrease in the numbers of labelled cells in more caudal area 23. When area 23c was replaced by area 31, label was still present in layer VI of the ventral bank of the cingulate sulcus. Approaching the splenium, the label seemed more evenly distributed across areas 29, 30, 23 and 31, though area 23a often appeared to have the highest concentration. This pattern continued to the caudal limit of the posterior cingulate cortex, where the numbers of labelled cells diminished.

The HRP injection in case ACy26 involved the anterior medial nucleus, but extended ventrally to include the midline nuclei including nucleus reuniens (Fig.9). Labelled cells were found across rostral area 23 in layer VI, with the highest concentration at the junction of areas 23b and 23c. The lower bank of the cingulate sulcus (area 23c) and area 23a contained appreciably fewer labelled cells, while areas 29 and 30 contained scarcely any label at rostral levels. This pattern shifted at more caudal levels (still in front of the splenium) so that more equivalent amounts of label were now found in areas 23a and 23b. Approaching the splenium, area 23a now typically had the highest concentration of label while area 31 contained scarcely any. The area 23a label now continued over the border so that for the first time there were also many labelled cells in area 30 (layer VI). Area 29 still appeared to contain almost no labelled cells. This obvious concentration of labelled cells in ventral 23a–area 30 continued caudal to the splenium, and included the ventral retrosplenial cortex.

A large injection of HRP (case ACy1) was centred in caudal anterior medial nucleus, but spread to reach the medial part of the anterior ventral nucleus and the rostral medial dorsal nucleus, along with midline nuclei including reuniens (Fig.9). Despite the involvement of additional nuclei, the pattern of cingulate label matched the other HRP cases with injections centred in the anterior medial nucleus (Table2). Like case ACy26, there was an increase in labelled cells in area 30 close to and behind the splenium. At some caudal levels, area 30 had the greatest density of label, i.e., more than area 23a. In contrast, area 23b showed a decrease in label going from rostral to caudal (as also found in ACy26).

In a third HRP case (ACy2) the anterior medial nucleus injection again reached the midline (Fig.9). This case showed low levels of HRP transport throughout the brain. There was a clear rostral–caudal posterior cingulate gradient (with most label in rostral areas) and any label was restricted to specific subareas, especially areas 23a and 23b (Table2).

There were three additional fluorescent tracer injections in the anterior medial nucleus (BRh3 left and right, BRh4 left; Fig.9), but in all three examples the same tracer was also injected into the medial dorsal nucleus in the opposite hemisphere. Evidence from those cases described previously strongly indicates that the medial dorsal thalamic nucleus receives extremely few crossed cingulate projections, and so the data from these three additional cases remain informative.

After DY was injected into the right anterior medial nucleus (BRh3), large numbers of labelled cells were seen in the ipsilateral hemisphere across rostral areas 23a, 23b, medial 23c and area 30, while label in area 29 was appreciably less frequent. Only small numbers of cells were seen in the deepest layer of area 31, close to the area 23 border. A notable feature was that labelled cell numbers decreased across the entire posterior cingulate–retrosplenial cortex when going more caudal. A similar rostral–caudal gradient was seen in the left hemisphere of case BRh4, which had an injection of DY in the mid and caudal anterior medial nucleus. Label was present in all posterior cingulate and retrosplenial areas rostral to the splenium, but was densest in areas 23a and 23b. The numbers of labelled cells decreased going more caudal, resulting in a more equal scattering of label across all areas. In the third injection (left hemisphere of BRh3), FB was injected into the mid and caudal anterior medial nucleus (Fig.9). Modest numbers of FB-labelled cells were consistently found in rostral areas 23a and 23b and in medial area 23c. Label was also present in area 30, sometimes at a concentration similar to that in adjacent area 23a. There was a relative decrease in label within area 29, with most of this label in area 29m. Approaching the level of the splenium, a consistent scattering of labelled cells was seen across the region of interest (including area 31), although there was a relative increase in labelled cells in areas 30 and 29 m. Around the splenium, area 30 appeared to contain the highest density of labelled cells.

The overall conclusion from the various fluorescent and HRP injections is that the anterior medial thalamic nucleus receives many inputs from the posterior cingulate and retrosplenial cortices, with area 23 and, to a lesser extent, area 30 providing the majority of these projections. In several cases there was a rostral–caudal gradient (with more label in rostral areas). The findings also showed lighter contralateral inputs to the anterior medial nucleus.

### Inputs to the anterior ventral thalamic nucleus

In two cases (BRh4 and BRh5) the tracer injections nuclei principally involved the anterior ventral thalamic nucleus (Fig.9). In each case, the animal received an injection of the same tracer in the opposite hemisphere.

In case BRh4 the tracer FB was injected into the anterior ventral nucleus, but probably extended into immediately adjacent parts of the anterior medial nucleus and paracentral nucleus. The FB injection in the opposite hemisphere was in the medial dorsal nucleus, which receives very few, if any, contralateral cingulate inputs (see earlier section). It is, therefore, assumed that ipsilateral FB label essentially reflects the anterior thalamic injection. The rostral posterior cingulate cortex contained labelled cells spread across areas 23d, then 23a and 23b and, to a lesser degree, area 23c. The highest concentration of cells was, however, in areas 29 and 30, and this was evident from the very rostral border. At more caudal levels this pattern became even more marked as areas 29 and 30 contained appreciably more labelled cells than area 23 (most in areas 30 and 29m, with areas 23a and 29l having the next highest concentration). The label in areas 29 and 30 appeared to include a few cells in layer V (in addition to those in layer VI). Light label was also found in layer VI of area 31. At the level of the splenium, the retrograde label was most concentrated in areas 30 and 29 m, although labelled cells were still present in area 23b (above and below the splenium) and in the prostriate area. Behind the splenium, there appeared to be more equal numbers of labelled cells across areas 29l, 29m, 30 and 23b.

In case BRh5, the left FB injection principally involved caudal parts of the anterior ventral nucleus, though it probably reached immediately adjacent parts of the anterior medial nucleus along with the rostral edge of the paracentral nucleus and nucleus centralis densocellularis. Although FB was also injected in the same case into the right laterodorsal nucleus (Fig.2) there appear to be very limited crossed inputs (see Fig.6) and so this case warrants description (Figs7 and 8). The FB label in the left hemisphere was strikingly similar to that described for the previous case (BRh4), except that there were even greater numbers of labelled cells in the posterior cingulate and retrosplenial cortices (Fig.8). Near the border with area 24, label was present in 23d, followed more caudally by large numbers of labelled cells across the deepest layers of rostral areas 29, 30 and 23 (Fig.7). Label in area 23c was the least frequent but, when present, was concentrated near the border with area 23b. Going more caudal, a great many labelled cells were seen in areas 29 and 30 (Figs7 and 8), contrasting with a clear relative reduction in areas 23a, 23b and 23c (though these 23 areas often contained substantial numbers of labelled cells). Approaching the splenium, the label was still concentrated in area 29 (especially 29l), with some label across layer VI of areas 23 and 31. Around the splenium the label became very heavily focussed in areas 29 and 30 (especially 29l), and this pattern continued caudal to the splenium (Figs7 and 8). Only a few cells in the ventral retrosplenial cortex were labelled. At the caudal limit of the posterior cingulate cortex, i.e. behind areas 29 and 30, labelled cells were scattered across the deep layer of area 23b (Fig.7).

**Figure 11:**
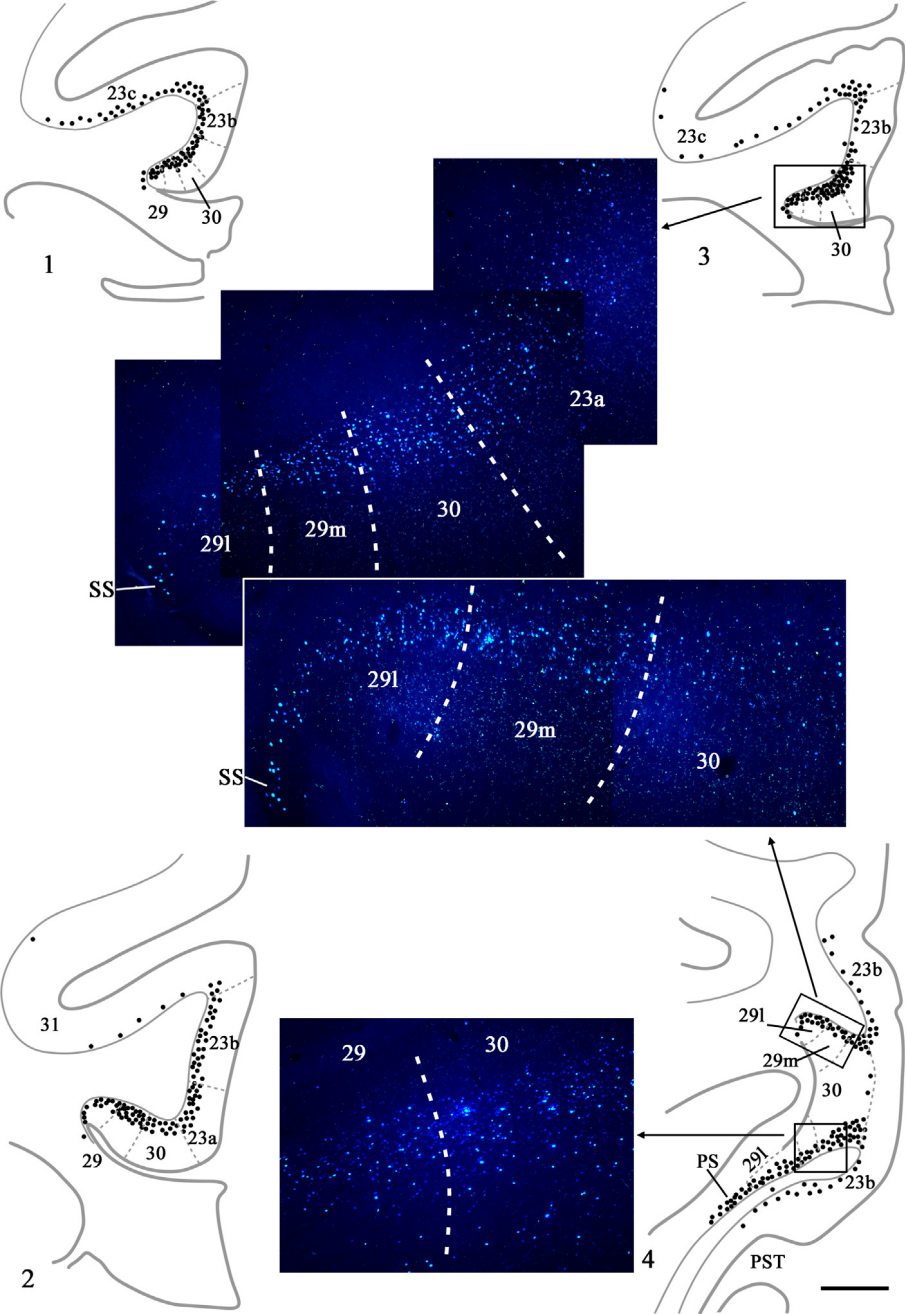
Drawings showing four coronal sections from case CT8C, which received a single injection of DY in the left anterior thalamus (anterior dorsal, anterior medial and anterior ventral nuclei). The dots reflect the relative distributions of labelled cells. The three areas in boxes are shown in the photomicrographs. In these photomicrographs the DY-labelled cells appear blue. PS, presubiculum; PST, prostriate cortex; SS, supracallosal subiculum. Scale bar, 2.0 mm.

In the final case (CT8C), an injection of DY in the left hemisphere was placed at the caudal junction of the anterior ventral nucleus, anterior dorsal nucleus and anterior medial nucleus (Fig.9), and potentially involved all three nuclei as well as nucleus centralis densocellularis. The pattern of cingulate label in this case appears to represent a combination of the inputs to the anterior thalamic nuclei. Numerous labelled cells were present across the posterior cingulate and retrosplenial areas (layer VI) producing a clear rostral–caudal gradient as caudal areas contained fewer labelled cells (Fig.11). At rostral levels, areas 29 and 30 consistently contained the largest number of labelled cells (up to 200 per section), although the adjacent area 23a also contained much label (Fig.11). Although areas 23b and 23c contained fewer labelled cells, there were still appreciable numbers, especially in rostral area 23 (where area 23c contained the least). Above the splenium, the numbers of cells in areas 29 and 30 remained similar to each other (∽100 per section in total) but, caudal to the splenium, area 29 now contained the most labelled cells (60–70 per section) while area 30 contained about half that number, with a further marked decrease in area 23 (Fig.11). At these very caudal levels, labelled cells were also present in the ventral retrosplenial cortex, and these cells were continuous with label extending dorsally from the presubiculum into the deepest layers of ventral areas 29 and 30 (Fig.11). In this same case (CT8C), a few labelled cells were present in the contralateral hemisphere (less than ten per section). As this case had a single injection site that did not reach the midline it was possible to be confident that this label reflected much lighter crossed projections that mirrored the distribution of ipsilateral label.

## Discussion

Retrograde tracers were placed into three thalamic sites, the anterior nuclei, the laterodorsal nucleus and the medial dorsal nucleus. Each site was injected in multiple cases, with the results showing clear agreement across repeat cases (Table2). The three target sites are linked, as pathology in each of these nuclei has been associated with memory loss (e.g., Markowitsch, 1982; Aggleton & Brown, 1999; Van der Werf *et al*., 2003; Edelstyn *et al*., 2006; Cipolotti *et al*., 2008; Carlesimo *et al*., 2011; Pergola *et al*., 2012), though there is debate over the respective roles of these nuclei, including their relative importance for familiarity and recollective based memory (Cipolotti *et al*., 2008; Aggleton *et al*., 2011; Pergola *et al*., 2012). One class of evidence used to support the notion that the medial dorsal and anterior thalamic nuclei make quite distinct contributions to memory relates to their markedly different patterns of inputs from the hippocampus (Aggleton, 2008).

In contrast to their medial temporal lobe inputs, the present study found clear similarities in the distribution of inputs to the anterior medial nucleus and medial dorsal nucleus (primarily from areas 23 and 30), although there were changes in density. This distribution contrasted with the pattern of inputs to the anterior ventral and laterodorsal nuclei (primarily from areas 29 and 30). Consequently, a key finding was the different pattern of results for the two adjacent anterior thalamic nuclei. These same results may help to explain the apparent difficulty of finding clear dissociations between the impact of anterior thalamic and medial dorsal nucleus damage on aspects of human memory (Cipolotti *et al*., 2008; Aggleton *et al*., 2011; Pergola *et al*., 2012). Furthermore, in view of evidence that the human retrosplenial cortex is important for episodic memory (Valenstein *et al*., 1987; Maguire, 2001; Vann *et al*., 2009), particular significance may be attached to the very dense inputs from areas 29 and 30 to both the anterior ventral and laterodorsal nuclei. These retrosplenial projections potentially enable the anterior ventral and laterodorsal nuclei to receive both direct and indirect hippocampal inputs, as the hippocampus projects densely to both thalamic nuclei as well as to the retrosplenial cortex but has much lighter projections to areas 23 and 31 (Rosene & Van Hoesen, 1977; Aggleton *et al*., 1986, 2012; Morris *et al*., 1999). In this way, the present results support the notion that the laterodorsal nucleus is important for memory, so strengthening the neuropsychological evidence, which is not yet clear-cut (Edelstyn *et al*., 2006). Although the present study provides the first unambiguous comparison between the thalamic projections of areas 29 and 30 within the primate retrosplenial cortex, the density of the thalamic projections from these cortical areas only changed gradually when going from one area to the next. While changes in the density of thalamic inputs arising from area 30 and adjacent area 23 were more apparent, this change was never abrupt. Finally, evidence was found that some projections from the primate posterior cingulate and retrosplenial cortices show a rostrocaudal topography, as previously indicated in rats and rabbits (Shibata, 1993, 1998; Shibata & Honda, 2012).

Of the thalamic targets, the medial dorsal nucleus is considered first as the results have particular implications for those cases with dual thalamic injections of the same tracer. Of the sites studied, the medial dorsal nucleus received the fewest posterior cingulate and retrosplenial inputs. The parvocellular portion appeared to be the preferred target, while inputs to the magnocellular part were extremely light. The cortical projections principally arose from layer VI, and mainly came from areas 23a and 23b (Table2). The findings from case CSR are consistent with the suggestion from anterograde tracer studies that the dorsolateral part of the medial dorsal nucleus might sometimes be a preferred target of area 23, though these inputs still remain light (Shibata & Yukie, 2003; see also Baleydier & Mauguiere, 1980; Yeterian & Pandya, 1988; Morris *et al*., 1999). The overall pattern of projections from the posterior cingulate cortex to the medial dorsal nucleus is mirrored by the light return projections, which target area 23, and area 23b in particular (Vogt *et al*., 1979; Baleydier & Mauguiere, 1980; Morris *et al*., 1999; Shibata & Yukie, 2003; Buckwalter *et al*., 2008). In marked contrast to its light posterior cingulate connections, the medial dorsal thalamic nucleus has dense reciprocal connections with the anterior cingulate cortex (Vogt *et al*., 1979, 1987; Baleydier & Mauguiere, 1980; Yeterian & Pandya, 1988).

A feature of the medial dorsal nucleus afferents was the almost complete absence of crossed cortical inputs. This finding supports the decision to maximise the data from two animals (BRh3 and BRh4) by placing two injections within the thalamus in different sites in opposite hemispheres, where one site was the medial dorsal nucleus (see Table1). This same issue extends to cases with other injection combinations (BRh5, BRh6). It is, therefore, relevant that Baleydier & Mauguiere (1980) reported how the cingulate (area 23) projections to the anterior ventral nucleus, medial pulvinar and lateral dorsal nucleus all remain ipsilateral. In agreement, our experiments indicate that any crossed projections to the lateral dorsal nucleus are extremely light (Fig.6). One exception is the anterior medial thalamic nucleus, which receives appreciable projections from contralateral areas 23 and 30 (Shibata & Yukie, 2003; see also cases BRh2 and CT8C).

Clear differences were found in the afferents to the target thalamic nuclei. Although the cingulate projections to the anterior medial thalamic nucleus showed general similarities with the distribution of projections to the medial dorsal thalamic nucleus, the inputs to the anterior medial nucleus were considerably more numerous. These anterior medial projections were, in turn, very different from those associated with injections in either the anterior ventral or laterodorsal thalamic nuclei. It was found that the inputs to the anterior medial nucleus were distributed across the posterior cingulate cortex, although there was typically a concentration in areas 23a and 23b, especially nearer their rostral borders. In contrast, area 29 contained appreciably less label. A very different pattern was seen for the anterior ventral and laterodorsal nuclei as now the very numerous projections principally arose from areas 29 and 30. With these differences in mind it is notable that, of the anterior thalamic nuclei, the anterior medial nucleus has the lightest projections to the posterior cingulate cortices (Vogt *et al*., 1979; Shibata & Yukie, 2003) and these projections preferentially target area 23 (Vogt *et al*., 1979, 1987), i.e. that area providing most of the projections to the anterior medial nucleus. This pattern of reciprocity also extends to the anterior ventral and laterodorsal nuclei as the source of most of their projections (areas 29 and 30) corresponds to the areas receiving most of the posterior cingulate gyrus projections from these thalamic nuclei (Vogt *et al*., 1987; Morris *et al*., 1999; Shibata & Yukie, 2003). For some cases there were rostral–caudal gradients, e.g., the area 23 inputs to the anterior medial nucleus were consistently most numerous in more rostral cingulate areas. Topographies within the posterior cingulate cortex have previously been described in other mammals, where the more rostral parts of the anterior thalamic nuclei are preferentially connected with caudal retrosplenial cortex, and *vice versa* (Shibata, 1993, 1998; Shibata & Honda, 2012). At the same time, we failed to see evidence of differential connections between the dorsal and ventral posterior area 23 with the anterior thalamic or medial dorsal nucleus, despite previous descriptions (Shibata & Yukie, 2003).

Consistent with the connectional findings for the primate brain, the anterior medial and anterior ventral nuclei of the rat thalamus have different properties. With respect to the retrosplenial cortex (the rat lacks posterior cingulate areas 23 and 31), the rat anterior medial nucleus is principally connected with the dysgranular cortex (equivalent to area 30) while the anterior ventral nucleus is densely interconnected with both the granular and dysgranular retrosplenial cortices (areas 29 and 30, respectively; Shibata, 1993; Van Groen *et al*., 1993; Van Groen & Wyss, 1990, 2003). This pattern clearly parallels the present findings for the primate brain. Furthermore, even when inputs to the rat anterior medial and anterior ventral thalamic nuclei arise from the same structure, they are either topographically distinct, e.g. in the mammillary bodies (Seki & Zyo, 1984; Shibata, 1992, 1998; Hopkins, 2005; Vann *et al*., 2007), or they show subtle localisation differences, with only rare examples of individual neurons that project to both anterior thalamic nuclei (Shibata, 1992, 1998; Aggleton *et al*., 2010; Wright *et al*., 2013). Electrophysiological studies in rats reinforce these differences as the anterior ventral thalamic nucleus contains a much higher proportion of cells that fire rhythmically with theta than found in the anterior medial nucleus (Vertes *et al*., 2001; Albo *et al*., 2003; Tsanov *et al*., 2011). Building on these properties it has been suggested that the anterior ventral nucleus provides a ‘return loop’ that brings together activity in the hippocampus with that in the retrosplenial cortex and the medial mammillary nucleus (Aggleton *et al*., 2010). In contrast, the primate anterior medial nucleus has the densest prefrontal connections of the anterior thalamic nuclei (Kievet & Kuypers, 1977; Xioa & Barbas, 2002a,b), connections that suggest a greater involvement in cognitive flexibility and executive functions.

The present study found that the laterodorsal and anterior ventral thalamic nuclei had very similar patterns of posterior cingulate and retrosplenial inputs. Tracer injections involving either nucleus revealed numerous layer VI projections that largely originated from the retrosplenial cortex (areas 29 and 30) although areas 23 and, to a lesser extent, area 31 contribute. Within much of area 23 there was a ventral–dorsal gradient with most inputs arising from area 23a. It has long been appreciated that the laterodorsal nucleus shares some of the general connectivity properties of the anterior thalamic nuclei, e.g., reciprocal connections with the hippocampal formation, though critically it differs in receiving few, if any, inputs from the mammillary bodies (Hopkins, 2005; Vann *et al*., 2007). In the rat brain, the laterodorsal nucleus contains head-direction cells. These cells show a greater firing rate when the rat is facing a particular direction (Mizumori & Williams, 1993; Taube, 2007). Head direction cells are also found in the anterior dorsal and anterior ventral thalamic nuclei (Taube, 2007; Tsanov *et al*., 2011). Given these shared electrophysiological properties it is significant that both area 29 and area 30 also contain head direction cells in the rat brain (Chen *et al*., 1994; Cho & Sharp, 2001) and that both cortical areas are densely interconnected with thalamic nuclei containing head direction cells (the laterodorsal and anterior ventral thalamic nuclei, along with the anterior dorsal nucleus). As a consequence, rodent areas 29 and 30, along with their thalamic connections, are assumed to be important for spatial learning and navigation (Sutherland & Hoesing, 1993; Mizumori *et al*., 2001), a function also linked to areas 29 and 30 in the human brain (Maguire, 2001; Epstein, 2008).

By virtue of their thalamic projections, the pyramidal cells in layer VI of the posterior cingulate and retrosplenial cortices take on a particular significance. The same layer gives rise to the anterior thalamic projections in the rat (Sripanidkulchai & Wyss, 1987; Van Groen *et al*., 1993), where disconnection studies have underlined the likely significance of these particular projections for spatial learning (Sutherland & Hoesing, 1993). The same layer VI cells that project to the rat anterior thalamus also receive intrinsic inputs from layer II (Amin *et al*., 2010), while layer VI also gives rise to intrinsic projections within retrosplenial cortex (Sripanidkulchai & Wyss, 1987) as well as projections to the anterior cingulate cortex and superior colliculus (Van Groen & Wyss, 1990, 2003). In the rabbit it has been found that units in layers V and VI of area 29 show training-induced activity changes in the early stages of learning, and that these training-induced changes precede those in more superficial layers of area 29 (Gabriel *et al*., 1980; Gabriel, 1993). Subsequent analyses based on electrophysiological changes in the anterior cingulate cortex, retrosplenial cortex and limbic thalamus led to the proposal that retrosplenial–thalamic connections mediate associative, i.e. acquired, attention in later stages of learning, while anterior cingulate cortices are more important at earlier stages of learning (Smith *et al*., 2002). That layer VI of the retrosplenial cortex projects to both the limbic thalamus and the anterior cingulate cortex would appear to further bind together these processes. As already noted, there is also much evidence that both the anterior thalamic nuclei and retrosplenial cortex are vital for normal episodic memory (Valenstein *et al*., 1987; Aggleton & Brown, 1999; Van der Werf *et al*., 2000, 2003; Maguire, 2001; Vann *et al*., 2009; Carlesimo *et al*., 2011). The repeated finding that both regions show abnormalities from almost the earliest stages of Alzheimer's disease (Braak & Braak, 1991a,b; Minoshima *et al*., 1997; Scahill *et al*., 2002; Nestor *et al*., 2003a,b; Buckner *et al*., 2005; Pengas *et al*., 2010) strongly implies that retrosplenial–thalamic interactions have broad, but important, effects upon cognition in health and disease.

## Abbreviations

DY: diamidino yellow
FB: fast blue
HRP: horseradish peroxidase
